# Cisgenic apple trees; development, characterization, and performance

**DOI:** 10.3389/fpls.2015.00286

**Published:** 2015-04-27

**Authors:** Frans A. Krens, Jan G. Schaart, Aranka M. van der Burgh, Iris E. M. Tinnenbroek-Capel, Remmelt Groenwold, Linda P. Kodde, Giovanni A. L. Broggini, Cesare Gessler, Henk J. Schouten

**Affiliations:** ^1^Wageningen UR Plant Breeding, Wageningen University and Research CentreWageningen, Netherlands; ^2^Plant Pathology, Institute of Integrative Biology, ETH ZürichZürich, Switzerland

**Keywords:** cisgenesis, apple, *Malus × domestica*, *MdMYB10*, anthocyanins, *Rvi6*, scab resistance, field trial

## Abstract

Two methods were developed for the generation of cisgenic apples. Both have been successfully applied producing trees. The first method avoids the use of any foreign selectable marker genes; only the gene-of-interest is integrated between the T-DNA border sequences. The second method makes use of recombinase-based marker excision. For the first method we used the *MdMYB10* gene from a red-fleshed apple coding for a transcription factor involved in regulating anthocyanin biosynthesis. Red plantlets were obtained and presence of the cisgene was confirmed. Plantlets were grafted and grown in a greenhouse. After 3 years, the first flowers appeared, showing red petals. Pollination led to production of red-fleshed cisgenic apples. The second method used the pM(arker)F(ree) vector system, introducing the scab resistance gene *Rvi6*, derived from apple. *Agrobacterium*-mediated transformation, followed by selection on kanamycin, produced genetically modified apple lines. Next, leaves from *in vitro* material were treated to activate the recombinase leading to excision of selection genes. Subsequently, the leaf explants were subjected to negative selection for marker-free plantlets by inducing regeneration on medium containing 5-fluorocytosine. After verification of the marker-free nature, the obtained plants were grafted onto rootstocks. Young trees from four cisgenic lines and one intragenic line, all containing *Rvi6*, were planted in an orchard. Appropriate controls were incorporated in this trial. We scored scab incidence for three consecutive years on leaves after inoculations with *Rvi6*-avirulent strains. One cisgenic line and the intragenic line performed as well as the resistant control. In 2014 trees started to overcome their juvenile character and formed flowers and fruits. The first results of scoring scab symptoms on apple fruits were obtained. Apple fruits from susceptible controls showed scab symptoms, while fruits from cisgenic and intragenic lines were free of scab.

## Introduction

In 2006, the concept of cisgenesis was brought to the attention of a large group of scientists and opened for debate by publications in Nature Biotechnology and EMBO Reports (Schouten et al., 2006a,b). The main reason for this was to explore possibilities to apply genetic modification (GM) as a tool to speed up breeding and to open up novel ways of breeding in crops such as apple in a way that would be acceptable to a wide public. There was and still is a great need for faster breeding tools, especially in woody plant species or others with a long generation time, and in vegetatively propagated heterozygous crops, e.g., apple, pear, potato, and tulip. Crosses with “wild” relatives require several rounds of back-crossing with the elite parent in order to get rid of undesired, negative traits originating from the wild parent. In apple, true back-crosses cannot be done because of self-incompatibility, adding to the complexity of apple breeding. So, once a high quality, elite variety has been bred after many years, a new crossing for the introduction of a single trait will make it disappear without a chance of ever being able to breed a similar variety again (Gardiner et al., 2007). Introduction of a single trait in an elite variety maintaining all good characteristics through GM, therefore, seems a perfect solution for these crops (Borejsza-Wysocka et al., 2010). However, suggested or perceived risks of GM has severely hampered large-scale applications of this technology in apple or other food crops. To accommodate to some of the major objections, with the aim of reducing as much as possible public concerns and biosafety issues, the concept of cisgenesis was formulated. Here, introduction of desired traits is still achieved by GM as a tool but the origin of the gene(s) is from sexually compatible plants, and the structure of the gene with native promoter, terminator and exons and introns is as it is present within the related donor plant. No foreign genes are present in the final product, particularly no antibiotic resistance genes of bacterial origin. Such crops were found to be more acceptable for consumers (Lusk and Sullivan, 2002; Gaskell et al., 2011). The European Food Safety Authority (EFSA Panel on Genetically Modified Organisms (GMO), 2012) compared the hazards associated with cisgenic, intragenic and conventionally bred plants and concluded that hazards associated with cisgenic plants are similar to the ones associated with conventionally bred plants.

The methods to generate cisgenic plants have been reviewed by Schaart et al. (2011). In short, three approaches can be used. One is to avoid the use of any selection genes of any other source than the recipient organism (e.g., apple) whatsoever. Second is to use two separate T-DNAs, one delivering the necessary selectable marker genes and the other carrying the genes-of-interest from cross-compatible species followed by segregation of these two T-DNAs in the progeny. Third is to use excision of selectable marker genes by inducing recombination (Schaart et al., 2004).

The generation of cisgenic crops is still very limited and has been reported in apple (Vanblaere et al., 2011, 2014), in barley (Holme et al., 2012), and in potato (Jo et al., 2014). In many crops the first steps toward the production of cisgenic plants are reported (Corredoira et al., 2012; An et al., 2013; Konagaya et al., 2013), e.g., in Rosaceae tree species such as pear (Righetti et al., 2014) and apple (Joshi et al., 2011; Würdig et al., 2013). In the latter crop, the first steps toward stacking multiple cisgenes and herewith multiple resistances have been taken (Gessler et al., 2014).

Here, we report on the development of new cisgenic apple lines by recombination-induced excision of non-apple gene sequences from the T-DNA of the transgenic lines described by Joshi et al. (2011). Induction was by dexamethasone treatment followed by selection on 5-fluorocytosine (Schaart et al., 2004). Those lines contained ultimately only the scab resistance gene *Rvi6* from *Malus floribunda* 821, either under control of its own promoter, just as the lines described by Vanblaere et al. (2011); Vanblaere et al. (2014) or under control of the promoter from the apple Rubisco gene, representing an example of an intragenic apple line. Micro-grafting first and later normal bud-grafting was used to multiply clonally all available lines to prepare for a field trial. Molecular screening confirmed the true cisgenic and intragenic nature of the plants, both the ones from Wageningen as well as the ones from Zürich, Switzerland. They were combined within one field-trial, established in the Netherlands, to study scab resistance levels in an orchard situation. In an earlier field trial using transgenic apples equipped with a barley hordothionin gene, evidence was found for prolonged scab resistance transferred by GM of elite apple cultivars (Krens et al., 2011). In that earlier trial, flowering was not permitted, but this time in the cisgenic orchard flowering and pollen dispersal were allowed. This enabled us to monitor fruit development and scab incidence on cisgenic apple fruits. With the results from this trial it was also possible to draw conclusions on long-term stability of the trait in the cisgenic apple lines.

Secondly, we report here on the generation of a new series of cisgenic apple lines using an entirely different approach. Using the apple MYB transcription factor gene, *MdMYB10*, carrying the natural rearrangement in the promoter as found in red-colored, red-fleshed apple varieties originating from var. “Niedzwetzkyana” (Espley et al., 2007, 2009), we constructed a minimal vector containing within the T-DNA borders only this *MdMYB10* gene, flanked by its native promoter and terminator. Earlier, Kortstee et al. (2011) reported on the suitability of a visual selection system using *MdMYB10* in producing transgenic apples. We describe here the production of red, cisgenic “Gala” and “Junami” apple lines selecting solely on anthocyanin production and red staining of callus, shoots, and plants.

## Materials and methods

### Construction of pMinMYB

**Figure 1:**
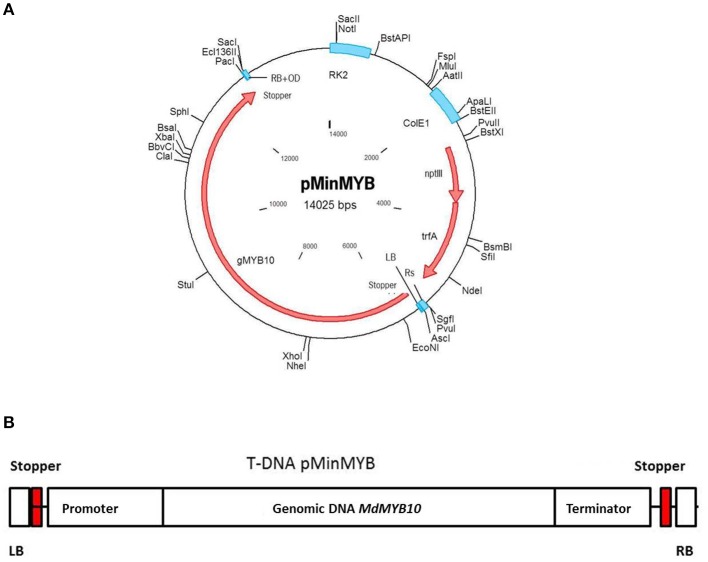
**Schematic representation of the pMinMYB vector carrying only the *MdMYB10* gene sequence between the T-DNA borders**. The *MdMYB10* sequence encompasses the promoter, 1768 bp including the additional repeats (6-fold repeat; R6) described by Espley et al. (2009), the coding region including introns 4184 bp and the terminator region of 1053 bp. **(A)** The entire plasmid. **(B)** The T-DNA region. RB, right border and LB, left border respectively; OD, overdrive sequence; Rs, recombination site.

Analogous to the construction of pMF1 (Schaart et al., 2011) the binary vector pBinplus (van Engelen et al., 1995) was used as starting point. Its backbone was combined with a T-DNA right border (RB) sequence supplemented externally with the overdrive sequence (Peralta et al., 1986). This intermediary vector also contained an Rs recombination site and *PacI/AscI* restriction sites between the RB and the Rs sequences for cloning. Thus, the Rs was located outside the T-DNA and has no function in this vector. The *MdMYB10* gene sequence including the natural mutation in the promoter (Espley et al., 2009) and terminator was isolated by PCR from the plasmid pART27.21, kindly provided by Dr. A.C. Allan of Plant and Food Research, Auckland, New Zealand, containing the genomic sequence of the red-fleshed cultivar “Red Field” (Espley et al., 2007). The forward primer of 63 nucleotides was designed to start with an *Asc*I restriction site followed by the LB sequence from the nopaline T37 Ti-plasmid T-DNA sequence (Schaart et al., 2011), a stopper sequence (CTAATTAACTAA)providing stop codons in all three reading frames (in both forward and reverse complement orientation) to prevent undesired read-through from within the T-DNA into the apple genome and finally the MYB10 sequence. The reverse primer (34-mer) started with a combined *PacI*/stopper sequence followed by the MYB10 sequence (Table S1, including cycling conditions). The amplified fragment of 7099 basepairs was cloned into pGEM-Teasy (Promega, Madison, USA) and later isolated again by digestion with *AscI/PacI*. The intermediary vector containing the RB was also opened with *AscI/PacI* and the *MdMYB10* fragment was ligated into it and subsequently transformed into *E.coli* XL10gold (Agilent Technologies Inc., Santa Clara, USA). After verification of the proper configuration and sequences the plasmid was isolated and transformed into AGL0 (Lazo et al., 1991) after which it was checked again. After this final verification the strain was used for transformation of apple. The final plasmid pMinMYB is presented in Figure 1A and the T-DNA in Figure 1B. Note that the non-functional Rs site is located outside of the T-DNA.

### Transformation and visual selection of pMinMYB regenerants

Transformation of apple explants was performed essentially as described by Puite and Schaart (1996) and Joshi et al. (2011). As explants, mostly leaf parts were taken but also meristem tips were chopped and exposed to *Agrobacterium*. Conditions were similar for the two types of tissues. Four different cultivars were used, i.e., “Gala,” “Junami,” “Wellant,” and “Mitchgla” (the latter being a “Gala” mutant). Selection was purely visual monitoring the occurrence of red, anthocyanin producing cells and callus parts. Red callus of sufficient size was isolated and cultured separately on elongation medium (SEM) and propagation medium (SPM), as described by Puite and Schaart (1996). Red shoots were collected and clonally multiplied for characterization and preparation for grafting.

### Molecular characterization I

From all shoots showing evidence of enhanced anthocyanin production DNA was isolated according to Doyle and Doyle (1987). All lines were tested using PCR for the presence of the mutated promoter in *MdMYB10*. The absence of *Agrobacterium* was confirmed by checking for *virG*; the absence of backbone sequences was monitored by doing a PCR on *trfA*. A final test was checking for the absence of *nptII*. Primer combinations are presented in Table S1 along with their respective thermal cycling conditions.

### Dexamethasone treatment and 5-FC selection

Transgenic “Gala” lines were produced earlier (Joshi et al., 2011). The constructs they used, were based on the pMF1vector for induced marker excision. pMF1 contains two recombination sites (Rs) and between those a Recombinase-LBD gene fusion and a dual positive/negative selection marker gene fusion are located (for further details, see Schaart et al., 2011). In the multiple cloning site of pMF1 Joshi et al. (2011) put the apple scab resistance gene *Rvi6* as gene-of-interest. They tested several promoters for monitoring efficacy in expression and disease resistance, i.e., different sizes of the *Rvi6* promoter itself (288 bp SP and 2000 bp LP; giving rise eventually to cisgenic lines) and the apple rubisco promoter (1600 bp; yielding intragenic lines). From the 13 transgenic “Gala” lines obtained by Joshi et al. (2011), still containing selectable marker genes, seven were subjected in this study to treatment with dexamethasone in order to induce marker removal and generate cisgenic or intragenic plants (see **Table 2**). For this, leaf explants were cut from the first four unfolded leaves from *in vitro* grown plantlets. The leaves were put in liquid SIM medium (Puite and Schaart, 1996) and cut transversely giving two cut edges per explant. All explants from each line were collected in small plastic containers in 25 ml liquid SIM supplemented with 50 μM dexamethasone (dex) and inoculated overnight for 17 h in the dark with slow shaking (100 rpm). After this, the explants were removed from the liquid medium, blotted dry, and placed with the abaxial side up on SIM supplemented with 500 mg/L 5-fluorocytosine (5-FC) and 1 μM dex with 20 explants/dish. After 4 weeks explants were transferred to fresh SIM medium without dex but still with 5-FC (500 mg/L). In all subsequent regeneration steps (Puite and Schaart, 1996) 500 mg/L 5-FC was included in the media. After establishment and confirmation of the cisgenic or intragenic nature of the regenerants further multiplication and preparation for grafting was done on medium without 5-FC.

### Molecular characterization II

Selected regenerants after dex treatment and 5-FC selection were individually propagated and from them DNA was isolated according to Doyle and Doyle (1987). PCR analyses were done using primer sets for *Rvi6, nptII, LBD*, and *nptIII*. These analyses should confirm presence of the scab resistance gene *Rvi6* and absence of *nptII* and *LBD* sequences. The latter would indicate that recombination has successfully removed the DNA between the Rs recombination sites (Schaart et al., 2004, 2011). With *nptIII* primers the absence of backbone sequences of the binary vector can be confirmed. For obtaining the permit for the field trial in the Netherlands the government required a multiplex PCR in which in one mix the presence of the *Rvi6* gene and the absence of the bacterial antibiotic resistance gene *nptIII* was demonstrated. For this, the two sets of primers were combined in one mix and amplification was performed on the samples. This was done for both the Dutch cisgenic and intragenic lines as well as for the Swiss cisgenic lines. Primer combinations are presented in Table S1 along with their respective cycling conditions.

### Micro-grafting and field trial organization

*In vitro* multiplied cisgenic lines, both from pMinMYB transformations and from *Rvi6* transformations were grafted on young apple seedlings as described by Joshi et al. (2011) and cultured in a greenhouse. In a later stage buds were collected from growing branches and grafted onto M9 rootstocks as described by Krens et al. (2011) and grown in a greenhouse until ready for transfer to the field (i.e., the *Rvi6* carrying cis- and intragenic lines). As controls *in vitro* propagated wild-type, scab susceptible “Gala” and wild-type scab-resistant “Santana” underwent an identical treatment of micro-grafting and grafting on M9. In October 2011 the trees were planted in a randomized block design in four rows as described earlier by Krens et al. (2011). In total, 25 trees were planted from the cisgenic Dutch line, SpVf2-11.1, the intragenic Dutch line, RbcVf2-11.D2, from the wild-type “Gala” and wild-type “Santana” controls and eight individual trees from each of three Swiss cisgenic lines, C7.1.49, C11.1.53, and C12.1.49 (Vanblaere et al., 2011, 2014). To avoid border effect eight commercially produced “Golden Delicious” trees grafted on M9 were planted at the north and south ends of the rows (four per row, two north and two south). In between as extra pollinators 12 other non-GM trees (four “Elstars,” scab susceptible, and eight from the Wageningen UR breeding program, scab resistant) were planted. In total, a number of 152 trees were part of the field trial. In 2014 flowering occurred and in April flowers were hand-pollinated using pollen collected from trees present at the Wageningen UR apple breeding location in Randwijk, the Netherlands. Natural pollination could also have occurred among the trees.

### Scab resistance assay

The plot where the trees were planted was the same as was used previously in a field trial from 2003 until 2008 with transgenic apple trees scoring for scab resistance after introduction of a barley hordothionin gene (Krens et al., 2011). In 2012 a natural infestation took place in the field trial. In 2013 and 2014 young leaves of the trees were inoculated artificially with a spore-suspension of an uncharacterized mixture of apple scab, *Venturia inaequalis*, isolates obtained from the breeding location in Randwijk. Inoculations and subsequent disease scoring were done as described earlier (Krens et al., 2011) using the semi-quantitative classification Vi-F-2 (King et al., 1998). Apple fruits were not separately inoculated but monitored for presence or absence of scab symptoms that arose spontaneously on the apples.

## Results

### *MdMYB10*, anthocyanin production

**Figure 2:**
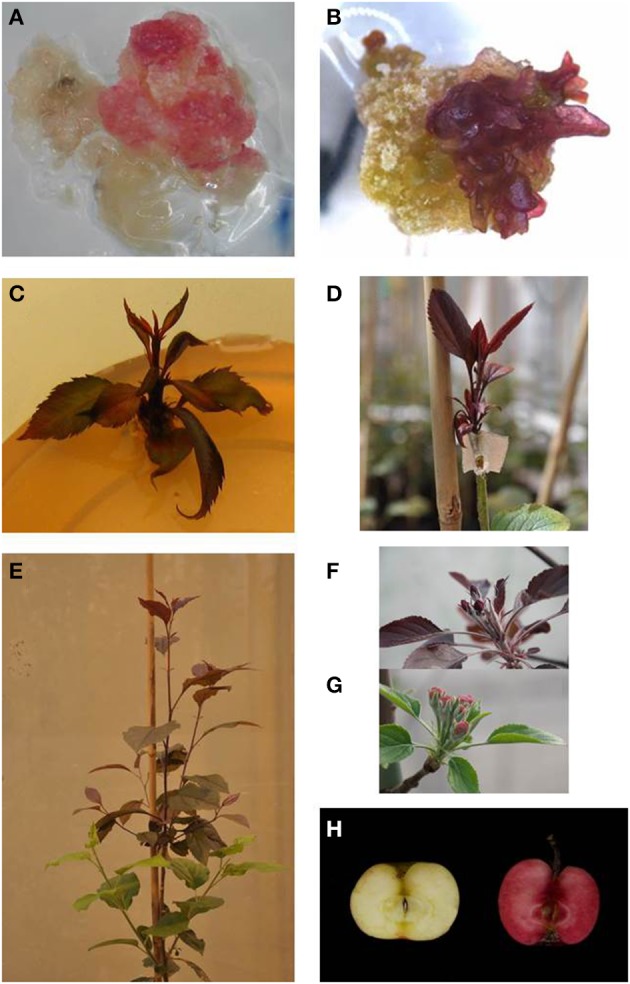
**Different steps in the process of generating cisgenic *MdMYB10* apple plants based on purely visual selection**. **(A)** Callus showing red coloration by anthocyanin production. **(B)** The onset of regenerating red shoots. **(C)** Red-colored shoots ready for propagation. **(D)** Micrografted, *in vitro* propagated red shoot. **(E)** Plant showing scion (top, red) and rootstock (bottom, green). **(F)** Developing red-colored flower buds on a cisgenic Junami line. **(G)** Wild-type Junami flowering. **(H)** Junami fruits, left: wild-type; right: *MdMYB10*-containing red-fleshed cisgenic apple.

**Figure 3:**
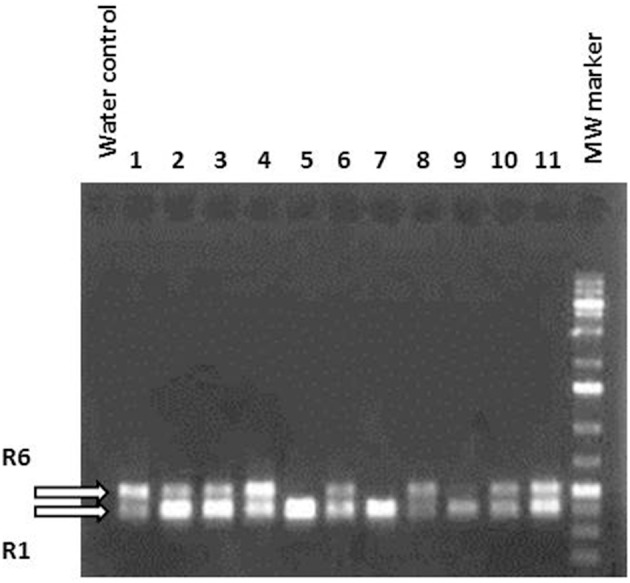
**PCR using primers located in the *MdMYB10* promoter region encompassing the microsatellite repeat domain R**. R1 represents the wild-type gene fragment and R6 the rearranged version being larger because of the six repeats. Lanes 1–11 show randomly chosen, individual regenerants with lanes 5, 7, and 9 having only one amplified fragment, i.e., the wild-type. These lines were green and prove to be not transformed. The others were considered potentially cisgenic.

Several independent transformation experiments were carried out with Agrobacterium AGL0(pMinMYB) for each of the four cultivars tested, “Gala” (*n* = 3), “Junami” (*n* = 3), “Mitchgla” (*n* = 6), and “Wellant” (*n* = 5). Many of them without any success. In the end, four red “Gala” shoots were obtained, three “Junami,” three “Mitchgla,” and zero “Wellant.” Table 1 shows the efficiencies calculated as the number of red shoots obtained divided by the total number of explants inoculated × 100%. The efficiencies of respectively, 0.55, 0.57, 0.12, and 0% for the aforementioned cultivars without selection were much lower when compared to transformation using a strain with a pMF1 based binary vector with the *MdMYB10* gene together with a *nptII* gene for selection on kanamycin (data not shown). Here, efficiencies were 13.8, 0.4, 4.3, and 0.22%, respectively. Figure 2 (A through H) shows the different steps in the generation of cisgenic plants based on visual selection for anthocyanin production. In Figure 3 the PCR results for the presence of the newly introduced *MdMYB10* gene are demonstrated. The primers were chosen to discriminate between the resident, wild-type promoter containing a single 23 bp minisatellite motif (R1) and the mutated promoter, containing six repeats of this motif (R6) (Espley et al., 2009). PCR showed size differences in the amplified fragments. In successfully transformed cisgenic plants both copies were present yielding two fragments in the gel.

**Table 1:** **Transformation efficiencies for the four cultivars tested pooled from multiple independent experiments without any selection other than by color**.

**Cultivar**	**Number of explants**	**Number of red calli**	**Number of red shoots**	**Transformation efficiency (%)**
Gala	730	6	4	0.55
Junami	530	3	3	0.57
Mitchgla	2570	4	3	0.17
Wellant	1420	4	0	0

### *Rvi6*, scab resistance

#### Production of cisgenic apple material

Seven GM apple lines produced earlier by Joshi et al. (2011) were subjected to a dex treatment and subsequent 5-FC selection to induce the activity of the introduced Recombinase gene and to remove by recombination all sequences between the two Rs sites within the T-DNA leading to marker-free and cisgenic or intragenic apple lines. The genotypes encompassed two lines carrying the long promoter construct (2000 bp, see Joshi et al., 2011), three lines with the short promoter and two lines with the apple Rubisco promoter (see Table 2).

**Table 2:** **Efficiency of the establishment of marker-free cis-/intragenic apple lines by dex treatment and 5-FC selection**.

**Line code**	**T-DNA copy number**	**Number of explants dexed**	**Number of explants giving callus**	**Number of explants giving shoots**	**Percentage regeneration**	**Number of propagated lines**	**Checked by PCR and grafted**	**To field**
LPVf2−1	2	113	0	0	0	−	−	−
LPVf2−4	1	187	0	0	0	−	−	−
SPVf2−2	1	245	167	3	1.2	0	−	−
SPVf2−11	2	123	123	8	6.5	2	2	1
SPVf2−15	1	88	0	0	0	−	−	−
RbcVf2−11	1	122	122	33	27	2	2	1
RbcVf2−12	2	274	0	0	0	−	−	−

*The lines are a selection of the transgenic lines reported by Joshi et al. (2011). Vf2 = Rvi6; LP, long promoter, 2000 bp; SP, short promoter, 288 bp and Rbc = apple rubisco promoter, 1600 bp*.

From the seven lines only three produced new regenerants from the leaves treated with dex, when grown on medium selective for successful excision events, i.e., with 5-FC. Two lines survived. Individual regenerants from these two lines were propagated and micrografted on seedlings after checking by PCR for the true cis- or intragenic nature. After a growth period in the greenhouse, 30 buds of each regenerant were grafted onto M9 rootstocks of which ultimately 25 plants were transferred to the field plot.

#### Molecular characterization of cisgenic and intragenic lines

**Figure 4:**
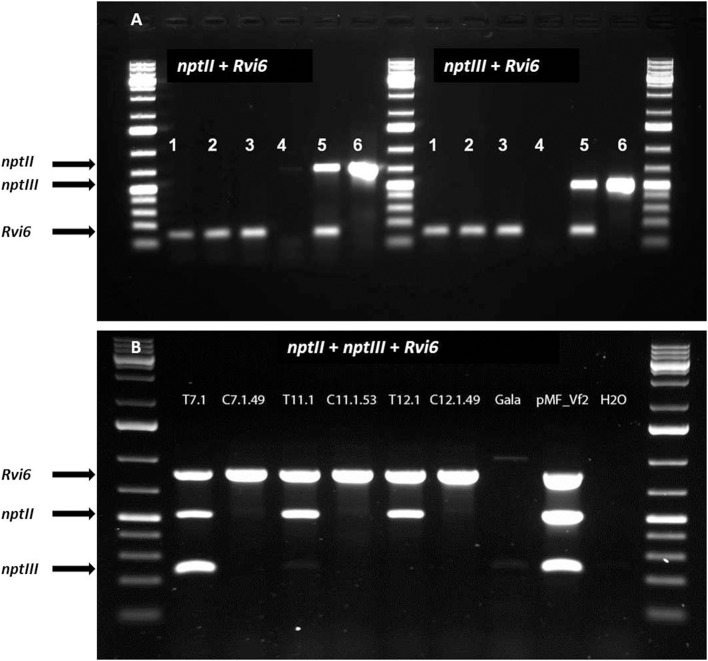
**Multiplex PCRs required by Dutch competent authorities in the application for a permit for the field-trial with the cisgenic and intragenic scab resistant apple lines**. The lines should be positive for the introduced gene-of-interest, *Rvi6*, but negative for the antibiotic resistance marker genes, *nptII* as plant selectable marker gene present between the border sequences before recombination and *nptIII* as bacterial selection gene indicator for backbone sequences. **(A)** Dutch intragenic and cisgenic lines. (1) RbcVf2-11D2, (2) RbcVf2-11D4, (3) SPVf2-11.1, 4. milliQ water control, (5) SPVf2-11 (parental line), (6) pMF1, the basic excision vector without *Rvi6*. **(B)** Swiss cisgenic and parental lines. T lines are the primary transformed parental lines; C lines are the derived marker-free, cisgenic lines. Gala is the non-GM control and pMF_Vf2, the plasmid used in transformation. Note the presence of backbone sequences in the parental lines SPVf2-11 and T7.1 and the absence of these sequences in the derived cisgenic lines, SPVf2-11.1 and C7.1.49, respectively.

In this study, we performed only PCR analyses. The parental lines were analyzed by Joshi et al. (2011) using Southern blot analysis and the T-DNA copy number in the lines before the dex treatment are given in Table 2. PCRs were done with primers for *Rvi6*, the scab resistance gene as gene-of-interest (should be present), for *LBD* and *nptII* representing the original selectable marker gene and the recombinase gene, present between the Rs sites (should be absent after excision) and for *trfA* and *nptIII* representing vector backbone gene sequences (should be absent). Dutch competent authorities required for granting permission for the field trial a multiplex PCR in which in one mix primers had to be combined for a gene supposed to be present and a gene whose absence was to be demonstrated. For this, we used a combination of two primer sets for *Rvi6* and *nptIII*, and *Rvi6* plus *nptII* for the Dutch lines and a combination of three primer sets, *Rvi6, nptII*, and *nptIII* for the Swiss lines (Figures 4A,B). Table 3 shows all PCR results for the Dutch lines. The characterization of the Swiss lines is described in detail by Vanblaere et al. (2014).

**Table 3:** **Pooled PCR results for the parental lines and the derived cis- and intragenic line**.

**Line**	**Dex**	***Rvi6***	***nptII***	**LBD**	***nptIII***	***trfA***
SPVf2−11	−	+	+	+	+(!)	n.t.
SPVf2−11.1	+	+	−	−	−	−
RbcVf2−11	−	+	+	+	−	−
RbcVf2−11.D2	+	+	−	−	−	−

The line SPVf2-11 stood out because it proved to have backbone sequences present in the integrated DNA. This original line also had two T-DNA copies as found by Southern analysis. The derived cisgenic regenerant line, SPVf2-11.1, proved to be without the backbone sequences and without the part between the Rs sites. The PCR pattern for the intragenic line, RbcVf2-11.D2 proved to be as expected.

#### Scab resistance

The trees were planted in 2011 and the first scoring for the severity of apple scab took place in 2012. In that year a spontaneous infection was observed and scoring was done in June when symptoms had spread throughout the orchard. Because also symptoms started to develop on the *Rvi6-*containing classically bred control cv. “Santana,” the strain of *Venturia inaequalis* was considered to be virulent for *Rvi6*. In view of the virulence, the field plot was treated with antifungal crop protectants to stop further spreading of this strain. This was done after scoring. The next year, 2013, no scab developed spontaneously and the trees were artificially inoculated with a mixture of scab isolates obtained from infected trees from our regular breeding site. Scoring was done 2–3 weeks later at two time-points by two different sets of persons. In 2013 a limited number of flower clusters were observed on two Swiss cisgenic trees, however, no fruits were obtained, despite pollination by hand.

In 2014, the last year reported here, after a very mild winter, the trees started growing and developing leaves in the second half of March and flowering occurred in April and in this month large-scale pollinations were carried out with wild-type pollen collected at the regular breeding site of Wageningen UR. End of May artificial inoculations were performed with a spore-suspension of a mix of *Rvi6-*avirulent strains, and approximately 3 weeks later scab severity was scored. Natural infection was also observed at the time of inoculation. Again, some virulence proved to be present among the *V. inaequalis* strains and fungicide spraying was initiated after scoring.

**Figure 5:**
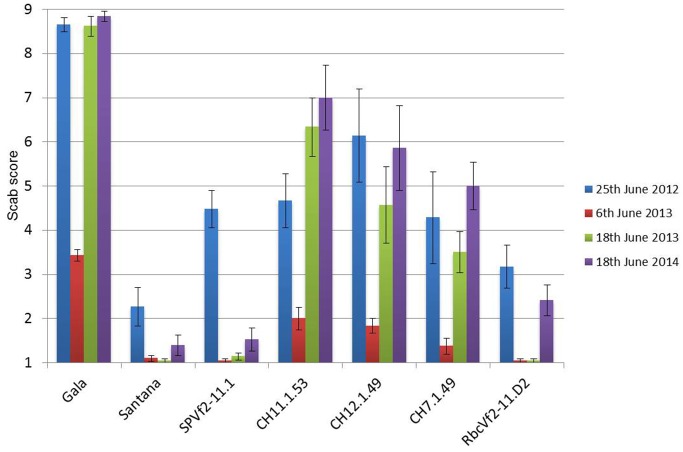
**A graphic representation of the accumulated scab disease scoring data of three consecutive years on the apple trees in the field**. In 2013 scoring was done at two moments because during the first scoring the disease had not sufficiently developed. Note progression of the disease in time. SPVf2-11.1, Dutch cisgenic line; RbcVf2-11.D2, Dutch intragenic line; CH codes represent the three Swiss cisgenic lines. Gala, wild-type susceptible control; Santana, wild-type resistant control. Scoring according to King et al. (1998) with 1 representing no symptoms and 2–9 increasing scab incidence. Bars present the standard deviation of means.

**Figure 6:**
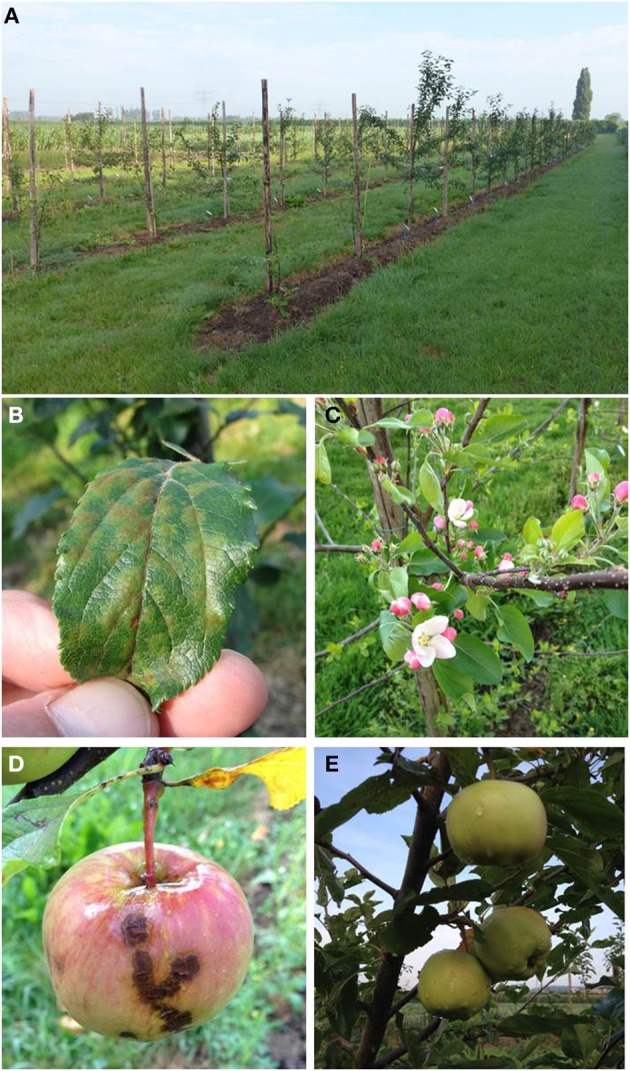
**An impression of phenotypes that were observed in the field trial**. **(A)** A general impression of the plot. **(B)** A diseased leaf of the susceptible wild-type cv. “Gala” after artificial inoculation with a *Venturia inaequalis* spore suspension. **(C)** Flowering of a cisgenic tree in 2014. Note the absence of scab symptoms on the leaves. **(D)** Scab symptoms on an apple fruit of one of the intragenic lines. **(E)** Unaffected apples on the cisgenic line SPVf2-11.1.

Over all 3 years and four scoring moments the same trend was found with respect to resistance, as shown in Figure 5. The non-GM resistant cv. “Santana” was equally resistant as the Dutch cisgenic line, SPVf2-11.1. The intragenic line, RbcVf2-11.D2 also scored very well, except for the last year, when it performed slightly worse than “Santana” and SPVf2-11.1. The wild-type susceptible control, “Gala,” proved indeed to be very susceptible and the Swiss cisgenic lines were intermediary with line C7.1.49 performing best. Figure 6 shows some of the phenotypes in the field.

Although the apple fruits were not treated separately with a spore-suspension of scab, symptoms did develop on the apples, especially on the ones on the wild-type controls (border trees and extra pollinator trees). All of the fruits on most of the cis- and intragenic lines (19 out of 21) were free of symptoms (Figure 6E) while two trees (intragenic) showed some symptoms on one of the many apples. The disease severity on the fruits was not be quantified this year.

## Discussion

### Visual selection, using *MdMYB10*

Generally in GM of plants, the cells that have taken up new DNA and have integrated it, are given a selective advantage in order to be able to discriminate and isolate them amongst the large majority of unchanged, wild-type cells. For this purpose selectable marker genes are used based on providing resistance to antibiotics or herbicides. These chemicals are added to the growth and regeneration media. Not using selectable marker genes will allow all cells to grow, divide, and regenerate into plantlets, and plants containing the insert will have to be identified amongst them by e.g., PCR. High transformation and regeneration rates are necessary for this strategy to work (Schaart et al., 2011), but successes have been reported in potato by De Vetten et al. (2003), in apple by Malnoy et al. (2007) and in wheat by Richardson et al. (2014). Kortstee et al. (2011) introduced the use of a visual marker, based on the apple *MdMYB10* gene, but the construct used still carried a kanamycin resistance gene within its T-DNA, and as such could not lead to cisgenic plants. Here, a binary vector was constructed having only the *MdMYB10* genomic sequence flanked by the stopper sequences within the T-DNA borders and nothing else. True cisgenic plants were obtained, by using only the red coloration induced by anthocyanin production as a means to visually identify cells, calli, and shoots that had taken up and integrated the apple cisgene. Transformation efficiencies expressed as the number of independent transformation events per number of explants inoculated with *Agrobacterium* ranged from 0 to 0.57%, depending on the cultivar used. These frequencies proved to be much lower than when using kanamycin as selectable agent, ranging from 0.22 to 13.8% (data not shown). Still, it proved feasible to produce cisgenic apple plants in this way, even with rather low transformation frequencies to start with. It has been observed that integration of the *MdMYB10* gene does not necessarily lead to expression and a red phenotype (Kortstee et al., 2011). Hence, the actual transformation frequency that was obtained in this study could have been higher. Silencing of the *MdMYB10* visual marker gene after integration would interfere with identification of cells or plants with successfully integrated T-DNA and subsequent selection, because of the lack of a phenotype. Only plants with an actively expressed newly introduced gene can be isolated, but this is true for any selection gene. An advantage of this system compared to the excision-based system used in the next part is that it avoids the use of a dex treatment step followed by a subsequent new regeneration step under 5-FC selection. Every extra step has its own efficiency and can lead to losses. Here, in a very direct way using *MdMYB10*, cisgenic plants are produced and selected even though frequencies are low. The data provide an indication of the frequencies that can be expected when no selection genes at all (cisgenic, visual or transgenic, chemical) are used in gene transfer experiments in apple. In case this type of making cisgenic apples is used for introduction of e.g., apple-derived resistance genes relying on regeneration and checking all regenerants by PCR, one can deduce how many explants to inoculate and how many plants to check based on the results presented here. Using *MdMYB10* as a cisgenic selection gene and combining it with traits such as diseases resistance might prove to be advantageous. However, taste should be unaffected and the consumers should broadly accept these red apple varieties. The cisgenic “Gala” and “Junami” lines can be used to investigate these aspects producing red-fleshed apple fruits with an appealing red appearance and an increased level of anthocyanin-based antioxidants. We applied for a permit for testing them in the field.

### Cisgenic scab resistant apples, using the recombination-based system pMF1

It was shown that using the recombination-based marker excision system based on our pMF1 vector series it was possible to generate a series of primary transformants (Joshi et al., 2011; Vanblaere et al., 2011; Righetti et al., 2014) starting with selection on kanamycin. In order to render these primary transformants cisgenic and remove the undesired genes from the T-DNA, activity of the recombinase enzyme needed to be induced by treatment with dex. This allowed selection for successfully removed foreign genes by growing the plant tissues on medium with the negative selection chemical 5-FC (Schaart et al., 2004, 2011). Success rates and frequencies have been shown here. From the seven lines that were subjected to the dex treatment, followed by selection on 5-FC regeneration medium, four gave no callus formation whatsoever and subsequently no regeneration of cisgenic shoots, independent of the construct used. Because the original lines were obtained from a regeneration process from explants after transformation, the potential for regeneration should be present. The reason for the lack of response could be that cells within the explants did not survive submersion in liquid for a prolonged period of 17 h. Another explanation could be that the dex treatment was ineffective and did not lead to excision. The continued presence of the *coda* gene (Schaart et al., 2004) would result in conversion of 5-FC into the toxic 5-fluorouracil (5-FU) inhibiting further growth and development. Also within the responding lines a great difference in the number of regenerants was observed, ranging from 1.2 to 27%, while the callusing response was much higher, often reaching 100%. As in all cases, the highly responsive cv. “Gala” was used, the differences must come from the fact that the lines represented different, independent transformation events. Also Vanblaere et al. (2011) and Righetti et al. (2014) reported variable success rates in obtaining cisgenic or marker-free plants after dex application and 5-FC selection.

We continued with propagating two regenerants from two lines (one cisgenic and one intragenic). The parental line (SPVf2-11) of the cisgenic (SPVf2-11.1) had two T-DNA copies (Joshi et al., 2011) and PCR analysis showed the presence of backbone sequences. Dex treatment resulted in removal of the *Recombinase* gene and the *nptII* gene, but also of the backbone sequences (*nptIII* and *trfA*). It can be hypothesized that the two T-DNA copies were integrated in tandem with a complete vector backbone in between. Recombination using the two outermost Rs sites would lead to one T-DNA copy without backbone and selection genes. This has not been confirmed yet, but a similar phenomenon was observed by Vanblaere et al. (2014) in one of their lines (T7.1), giving rise to cisgenic line (C7.1.49). No special effects were observed for line RbcVf2-11, it showed a high callusing response, a high regeneration rate and the expected PCR profile.

The Swiss parental and cisgenic lines and the Dutch parental lines were extensively studied at the molecular level by Southerns, PCR and expression level (Joshi et al., 2011; Vanblaere et al., 2014). Joshi et al. (2011) found no correlation between gene expression and T-DNA copy number and between expression and scab resistance in greenhouse assays while Vanblaere et al. (2014) found similar RNA levels before and after excision in two of the three lines studied. They also showed that expression of *Rvi6* in the cisgenic lines was not as high as in the resistant control “Florina” and also resistance was intermediary between “Florina” and the wild-type susceptible “Gala.” RNA-levels were not tested in the Dutch cis- and intragenic lines after dex treatment, but expression was 0.84× “Santana” for parental SPVf2-11 and 78× “Santana” in parental RbcVf2-11 (Joshi et al., 2011).

The resistance levels in the field proved to concur with the resistance levels in the greenhouse. This was found for the Swiss cisgenic lines and for the Dutch lines comparing the levels before dex-treatment in the greenhouse to the level after dex-treatment in the field (cisgenic). In the field, the cisgenic line SPVf2-11.1 performed equally well as “Santana.” The intragenic line RbcVf2-11.D2 also performed equally well as “Santana,” except for the last year. The Swiss cisgenic lines were intermediary with C7.1.49 giving the best results. A possible explanation for the difference in resistance among the four cisgenic lines could be that in the three Swiss lines a shorter promoter was used giving lower expression at the RNA level and lower resistance. The results from this study indicated that the introduced *Rvi6* gene was not only expressed at the RNA level, but also provided scab resistance in the field after propagation, grafting, growing, and planting. The phenotype proved to be stable over a period of 3 years. The observations made with the spontaneously arising virulent strain of *V. inaequalis*, giving symptoms on the conventionally bred, resistant “Santana” as well as on the cis- and intragenic lines, suggested that the resistance spectrum of the *Rvi6* gene in the field is similar in “Santana” and in the lines in which it was introduced using GM techniques. This confirmed what was found earlier by Joshi et al. (2011), who tested six avirulent and virulent monoconidial isolates of *V. inaequalis* on the parental lines in the greenhouse. This demonstrates the necessity to preferably stack multiple resistance genes. Cisgenesis can be an important instrument in achieving this. Finally, the first indications were obtained that the *Rvi6* gene is also active in the fruits in the cis- and intragenic lines. This will be studied in greater detail the coming years.

## Conclusions

Cisgenic apples can be obtained using only a visual marker for selection, even though transformation and regeneration frequencies are low. *MdMYB10* can be used as the sole selection marker. Cisgenic apples can also be made using a recombinase-based marker excision system. The efficiency of the excision and of the following regeneration under 5-FC selection varies from one line to the other, presumably depending on the transformation event. Optimization is required for each crop system. The introduced cisgene *Rvi6* is stable in phenotype over multiple years and performs similar to the gene in a natural configuration with respect to resistance level, resistance spectrum, and plant organs. It opens up the possibility to add new traits to elite apple cultivars maintaining the quality traits of the cultivar. This paper contributes to the accumulating evidence that cisgenic crops are similar to conventionally bred crops, as was already concluded for hazards associated to both breeding methods by EFSA Panel on Genetically Modified Organisms (GMO) (2012).

## Author contributions

Experiments were designed by FK, JS, GB, CG, and HS. Constructs were made by JS and AB. Transformations were done by AB and IT. Molecular analyses were performed by JS, AB, and LK. Scab evaluations were done by RG, FK, GB, CG, and HS. Field trial was prepared by FK and the Swiss material was analyzed and made available by GB and CG. Statistical analysis was done by HS and the draft manuscript was prepared by FK and revised by JS, HS, GB, and CG. All authors read and approved the final manuscript.

### Conflict of interest statement

The authors declare that the research was conducted in the absence of any commercial or financial relationships that could be construed as a potential conflict of interest.

## Abbreviations

SIM: shoot induction medium
*nptII(I)*: neomycinphosphotransferaseII(I)
*LBD*: ligand-binding domain
*trfA*: gene encoding a replication initiation protein of the broad host-range plasmid RK2.

## References

[B1] An C. F. Orbovic V. Mou Z. L. (2013). An efficient intragenic vector for generating intragenic and cisgenic plants in citrus. Am. J. Plant Sci. 4, 2131–2137 10.4236/ajps.2013.411265

[B2] Borejsza-Wysocka E. Norelli J. L. Aldwinckle H. S. Malnoy M. (2010). Stable expression and phenotypic impact of *attacin E* transgene in orchard grown apple trees over a 12 year period. BMC Biotechnol. 10:41. 10.1186/1472-6750-10-41 20525262 PMC2910661

[B3] Corredoira E. Valladares S. Allona I. Aragoncillo C. Vieitez A. M. Ballester A. (2012). Genetic transformation of European chestnut somatic embryos with a native thaumatin-like protein (*CsTL1*) gene isolated from *Castanea sativa* seeds. Tree Physiol. 32, 1389–1402. 10.1093/treephys/tps098 23086811

[B4] De Vetten N. Wolters A. M. Raemakers K. Van der Meer I. ter Stege R. Heeres E.. (2003). A transformation method for obtaining marker-free plants of a cross-pollinating and vegetatively propagated crop. Nat. Biotechnol. 21, 439–442. 10.1038/nbt801 12627169

[B5] Doyle J. J. Doyle J. L. (1987). A rapid DNA isolation procedure from small quantities of fresh leaf tissues. Phytochem. Bull. 19, 11–15.

[B6] EFSA Panel on Genetically Modified Organisms (GMO). (2012). Scientific opinion addressing the safety assessment of plants developed through cisgenesis and intragenesis. EFSA J. 10:2561 10.2903/j.efsa.2012.2561

[B7] Espley R. V. Brendolise C. Chagne D. Kutty-Amma S. Green S. Volz R.. (2009). Multiple repeats of a promoter segment causes transcription factor autoregulation in red apples. Plant Cell 21, 168–183. 10.1105/tpc.108.059329 19151225 PMC2648084

[B8] Espley R. V. Hellens R. P. Putterill J. Stevenson D. E. Kutty-Amma S. Allan A. C. (2007). Red colouration in apple fruit is due to the activity of the MYB transcription factor, MdMYB10. Plant J. 49, 414–427. 10.1111/j.1365-313X.2006.02964.x 17181777 PMC1865000

[B9] Gardiner S. E. Bus V. G. M. Rusholme R. L. Chagné D. Rikkerink E. H. A. (2007). Apple, in Genome Mapping and Molecular Breeding in Plant. Vol. 4, Fruits and Nuts, ed Kole C. (Berlin; Heidelberg: Springer-Verlag), 1–62.

[B10] Gaskell G. Allansdottir A. Allum N. Castro P. Esmer Y. Fischler C.. (2011). The 2010 eurobarometer on the life sciences. Nat. Biotechnol. 29, 113–114. 10.1038/nbt.1771 21301431

[B11] Gessler C. Vanblaere T. Parravicini G. Broggini G. A. L. (2014). Cisgenic ‘Gala’ containing the scab resistance gene from *Malus floribunda* 821 and the fire blight resistance genes from *M*. ‘Evereste’. Acta Horticult. 1048, 43–50.

[B12] Holme I. B. Dionisio G. Brinch-Pedersen H. Wendt T. Madsen C. K. Vincze E.. (2012). Cisgenic barley with improved phytase activity. Plant Biotechnol. J. 10, 237–247. 10.1111/j.1467-7652.2011.00660.x 21955685

[B13] Jo K.-R. Kim C.-J. Kim S.-J. Kim T.-Y. Bergervoet M. Jongsma M. A.. (2014). Development of late blight resistant potatoes by cisgene stacking. BMC Biotechnol. 14:50. 10.1186/1472-6750-14-50 24885731 PMC4075930

[B14] Joshi S. G. Schaart J. G. Groenwold R. Jacobsen E. Schouten H. J. Krens F. A. (2011). Functional analysis and expression profiling of *HcfVf1* and *HcrVf2* for development of scab resistant cisgenic and intragenic apples. Plant Mol. Biol. 75, 579–591. 10.1007/s11103-011-9749-1 21293908 PMC3057008

[B15] King G. J. Alston F. H. Brown L. M. Chevreau E. Evans K. M. Dunemann F. (1998). Multiple field and glasshouse assessments increase the reliability of linkage mapping of the *Vf* source of scab resistance in apple. Theor. Appl. Genet. 96, 699–708 10.1007/s001220050791

[B16] Konagaya K. Tsuda M. Okuzaki A. Ando S. Tabei Y. (2013). Application of the acetolactate synthase gene as a cisgenic selectable marker for *Agrobacterium*-mediated transformation in Chinese cabbage (*Brassica rapa* ssp. pekinensis). Plant Biotechnol. 30, 125–133 10.5511/plantbiotechnology.13.0124a

[B17] Kortstee A. J. Khan S. A. Helderman C. Trindade L. M. Wu Y. Visser R. G. F.. (2011). Anthocyanin production as a potential visual selection marker during plant transformation. Transgenic Res. 20, 1253–1264. 10.1007/s11248-011-9490-1 21340526 PMC3210953

[B18] Krens F. A. Schaart J. G. Groenwold R. Walraven A. E. J. Hesselink T. Thissen J. T. N. M. (2011). Performance and long-term stability of the barley hordothionin gene in multiple transgenic apple lines. Transgenic Res. 20, 1113–1123. 10.1007/s11248-011-9484-z 21243525 PMC3174370

[B19] Lazo G. R. Stein P. A. Ludwig R. A. (1991). A DNA transformation-competent *Arabidopsis* genomic library in *Agrobacterium*. Nat. Biotechnol. 9, 963–967. 10.1038/nbt1091-963 1368724

[B20] Lusk J. L. Sullivan P. (2002). Consumer acceptance of genetically modified foods. Food Technol. 56, 32–37.

[B21] Malnoy M. Borejsza-Wysocka E. E. Abbott P. Lewis S. Norelli J. L. Flaishman M. (2007). Genetic transformation of apple without use of a selectable marker. Acta Horticult. 738, 319–322.

[B22] Peralta E. G. Hellmiss R. Ream W. (1986). Overdrive, a T-DNA transmission enhancer on the *A. tumefaciens* tumour-inducing plasmid. EMBO J. 5, 1137–1142. 15966101 10.1002/j.1460-2075.1986.tb04338.x PMC1166919

[B23] Puite K. J. Schaart J. G. (1996). Genetic modification of the commercial apple cultivars Gala, Golden Delicious and Elstar via an *Agrobacterium tumefaciens*-mediated transformation method. Plant Sci. 119, 125–133 10.1016/0168-9452(96)04448-2

[B24] Richardson T. Thistleton J. Higgins T. J. Howitt C. Ayliffe M. (2014). Efficient *Agrobacterium* transformation of elite wheat germplasm without selection. Plant Cell Tissue Organ Cult. 119, 647–659 10.1007/s11240-014-0564-7

[B25] Righetti L. Djennane S. Berthelot P. Cournol R. Wilmot N. Loridon K. (2014). Elimination of the *nptII* marker gene in transgenic apple and pear with a chemically inducible R/Rs recombinase. Plant Cell Tissue Organ Cult. 117, 335–348 10.1007/s11240-014-0443-2

[B26] Schaart J. G. Krens F. A. Pelgrom K. T. B. Mendes O. Rouwendal G. J. A. (2004). Effective production of marker-free transgenic strawberry plant using inducible site-specific recombination and a bifunctional selectable marker gene. Plant Biotechnol. J. 2, 233–240. 10.1111/j.1467-7652.2004.00067.x 17147614

[B27] Schaart J. G. Krens F. A. Wolters A.-M. Visser R. G. F. (2011). Chapter 24, Transformation methods for obtaining marker-free genetically modified plants, in Plant Transformation Technologies, eds Steward C. N. Jr Touraev A. Citovsky V. Tzfira T. (Ames, IA: Wiley-Blackwell Publishing), 229–242.

[B28] Schouten H. J. Krens F. A. Jacobsen E. (2006a). Cisgenic plants are similar to traditionally bred plants. EMBO Rep. 7, 750–753. 10.1038/sj.embor.7400769 16880817 PMC1525145

[B29] Schouten H. J. Krens F. A. Jacobsen E. (2006b). Do cisgenic plants warrant less stringent oversight? Nat. Biotechnol. 24:753. 10.1038/nbt0706-753 16841052

[B30] Vanblaere T. Flachowsky H. Gessler C. Broggini G. A. L. (2014). Molecular characterization of cisgenic lines of apple ‘Gala’ carrying the *Rvi6* scab resistance gene. Plant Biotechnol. J. 12, 2–9. 10.1111/pbi.12110 23998808

[B31] Vanblaere T. Szankowski I. Schaart J. Schouten H. Flachowsky H. Broggini G. A. L.. (2011). The development of a cisgenic apple plant. J. Biotechnol. 154, 304–311. 10.1016/j.jbiotec.2011.05.013 21663775

[B32] van Engelen F. A. Molthoff J. W. Conner A. J. Nap J. P. Pereira A. Stiekema W. J. (1995). pBINPLUS: an improved plant transformation vector based on pBIN19. Transgenic Res. 4, 288–290. 10.1007/BF01969123 7655517

[B33] Würdig J. Flachowsky H. Hanke M.-V. (2013). Studies on heat-shock induction and transgene expression in order to optimize the Flp/*FRT* recombinase system in apple (*Malus × domestica* Borkh.). Plant Cell Tissue Organ Cult. 115, 457–467 10.1007/s11240-013-0376-1

