# Supplementary material for: Cisgenic apple trees; development, characterization, and performance
Source: Front Plant Sci. 2015 Apr 27;6:286. doi: 10.3389/fpls.2015.00286 (PMC4410516; doi:10.3389/fpls.2015.00286)
Supplement: Supplementary file 1 [file Table1.DOCX]

Table S1. Primers combinations and cycling profiles used in cloning and molecular analyses of the cisgenic apple lines.
